# Xenotransplantation—reflections on the bioethics

**DOI:** 10.1002/hcs2.18

**Published:** 2022-10-12

**Authors:** Xiaomei Zhai

**Affiliations:** ^1^ School of Population Medicine and Public Health Peking Union Medical College Dongcheng‐qu China

**Keywords:** xenotransplantation, organs, bioethics

## Abstract

**Background:**

Similar to most countries in the world, China has a severe shortage of human organs, and this is one of the main issues restricting the application of organ transplantation technology. In 2019 alone, only 19,454 (23.90%) of the 81,410 people waiting were able to receive organ transplants. There is an increasing focus from both the medical profession and society on how to fill the gap between supply and demand.

**Methods:**

Xenotransplantation using animal organs is being considered as one option to make up for the shortage of human organs for transplantation. For some years now, the international medical community has been examining the possibility of using animal organs for human transplant. However, the research has faced two important types of challenges: scientific and ethical issues.

**Results:**

In January 2021, the first clinical trial of transgenic pig heart transplantation into a human recipient was completed by the Medical Center of the University of Maryland in the United States. This has stimulated enthusiasm and interest in xenotransplantation.

**Conclusions:**

The trend towards xenotransplantation has highlighted global problems such as the severe shortage of organ transplant donors and the high cost of organ transplantation. China needs to consider how to cope with the scientific, public health, and social ethics challenges of xenotransplantation clinical trials.

## Graphical abstract

Xenotransplantation is being considered as one option to make up for the shortage of human organs for transplantation. However, it faces some serious ethical challenges.

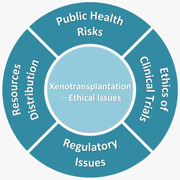

**Abbreviations**
CRT: Campaign for Responsible Transplantation
FDA: Food and Drugs Agency

## 1 INTRODUCTION

Similar to most countries in the world [1, 2], there is a severe shortage of human organs for transplantation in China. This is still one of the main issues restricting the application of organ transplantation technology. In 2019 alone, only 19,454 (23.90%) of the 81,410 people waiting were able to receive organ transplants [3]. There is an increasing focus among both the medical profession and society more generally on how to fill the gap between supply and demand.

Possible alternatives to human organs include mechanical devices, tissue engineering techniques, and the use of organs from animals (xenotransplantation). Mechanical devices are unsatisfactory because of unresolved risks, such as infection and the risk of blood clotting in patients. Post‐transplant patients require lifelong anticoagulant therapy, increasing the risk of bleeding in women during pregnancy and childbirth. It is also difficult for mechanical devices to truly replace organs with complex biochemical functions, such as the liver. This limits the options for the use of this technique. Tissue engineering technology is the use of living cells to create replacement organs and tissues. This is a promising technology, but it is still in an early stage of development. The development and employment of bioengineered functional organs are not realistic in the near future. Xenotransplantation is another option to make up for the shortage of human organs for transplant. For the past few years, the international medical community has been considering the possibility of using animal organs as human transplant donors, but the research has always faced scientific and ethical challenges. In January 2021, the first transgenic pig heart transplantation in human history was completed by the Medical Center of the University of Maryland in the United States. This further stimulated enthusiasm and interest in xenotransplantation. The trend toward xenotransplantation has highlighted global problems such as the severe shortage of organ transplant donors and the high cost of organ transplantation. However, there are still scientific, public health, and social ethics challenges to xenotransplantation, and any country considering this option will need to address these.

## 2 EARLY DISCUSSIONS ABOUT XENOTRANSPLANTATION

During the 1990s, there was an extensive discussion of xenotransplantation in the scientific literature, covering scientific and ethical issues raised by this technology. Authoritative medical journals, such as *JAMA*, *BMJ*, *The Lancet*, *Xenotransplantation*, and *Transplantation Proceedings*, published reviews of research and discussions of related issues [4, 5, 6, 9, 10]. Authoritative journals of medical ethics and bioethics, such as *The Hastings Center Report*, *Journal of Kennedy Institute of Ethics*, *Journal of Medical Ethics*, and *Journal of Medicine and Philosophy* also published articles on the ethical issues. The issue 391(6665) of *Nature*, published on January 22, 1998, contained an extensive discussion on xenotransplantation. Experts from different disciplines expressed concerns about the development of xenotransplantation technology and criticized biotechnology companies for the development of overly optimistic estimates. They emphasized that more basic research should be carried out, and recommended that clinical trials of xenotransplantation should be suspended [9]. Professor Fritz H. Bach, an authority on xenotransplantation at Harvard Medical School, opined that helping individuals relieve pain was an adequate justification for the risk to the public, which was known to exist but could not be quantified at the time. The consensus was that xenotransplantation is ultimately an ethical issue rather than a technical one [11]. At about that time, many countries around the world began to consider the ethical governance and legal issues around xenotransplantation.

China was late in starting xenotransplantation research. Some medical universities have performed xenotransplantation in animal models and have carried out related preclinical research. Some of these studies have been supported by major national projects in China. The number of articles on preclinical studies of xenotransplantation published in the journal *China Organ Transplantation* is increasing each year, as is the number of articles on xenotransplantation considered at the annual National Organ Transplantation Academic Conference. Nevertheless, ethical issues are hardly discussed.

## 3 CURRENT DISCUSSION OF THE ETHICS OF XENOTRANSPLANTATION

Xenotransplantation raises a number of ethical issues, and it is impossible to address all of them here. First, there are important issues related to animal welfare and animal rights. Most people agree that it is at least sometimes justified to use animals to improve human welfare, but we also agree that we should minimize animal suffering, and that there are relevant differences between animal species. For example, most people reject using nonhuman primates to produce organs for human transplantation. However, the use of pigs is considered more acceptable. There are also deep philosophical questions about what it means to be human, and how the use of animal organs and tissue affect our understanding of human dignity. This is particularly important for research dealing with brain tissue, but has also been raised for other types of research using stem cells that may develop into brain cells. I shall not cover any of these issues in my discussion here. Instead, I shall try to deal with four main questions and issues.

First, do the risks of a procedure justify the social benefits? The risks and benefits are mainly to the individuals undergoing transplantation. However, social costs and risks may also need to be considered, especially public health risks associated with the risk of infections.

Second, are the risks to individuals receiving xenotransplants justified by the potential benefits for them? For ordinary clinical procedures, risks are acceptable because they are outweighed by the potential benefits of the procedures. For experimental procedures such as xenotransplantation, we should use standards developed for clinical trials, specifically phase I or first in human‐type trials.

Third, given the public health risks and the special nature of clinical trials on xenotransplantation, should we consider special, more stringent, and comprehensive regulatory frameworks than for ordinary clinical procedures?

Finally, there are wider social policy issues of xenotransplantation that should be considered. For example, are resources distributed fairly and equitably, and do we need to take into account deeply held public attitudes? We should not automatically reject policies because they go against cultural or social attitudes. However, we do need to take attitudes into account when proposing policies, either by accepting that policies cannot be implemented because of such beliefs, or by attempting to change beliefs that are based on misunderstandings or false information.

## 4 PUBLIC HEALTH RISKS

In the late 1990s there were serious concerns about public health risks associated with xenotransplantation, especially the transmission of animal infections to humans. This included both the risk of infecting the transplant recipients themselves, and that infections originating in animals could mutate in an individual with a weakened immune system, placing other individuals at risk. In the worst‐case scenario, this could lead to pandemics. Some commentators called for a moratorium on all xenotransplantation until these concerns could be addressed [12]. However, most official reports called for a careful development of xenotransplantation with strong oversight mechanisms, and stringent quality controls at all stages of development. Examples of these reports include the 1996 Nuffield Council's report on xenotransplantation [12], the 1996 report of the US National Academies [13], and a WHO Consultation on Xenotransplantation in 1997 [14]. All of these groups highlighted the risk of disease transmission, and recommended that national authorities should develop national guidelines and/or regulations that would set standards and rules for xenotransplantation activities. There was some disagreement about whether there should be national review and approval of all xenotransplantation activities because of the special nature of the risks. Some argued that local ethics reviews would be sufficient. However, even when this was deemed sufficient, local review committees were expected to consult experts, and always follow national guidelines or regulations. These reports also recommended setting up national databases of transplant recipients so that they could be followed and monitored systematically for the rest of their lives for any infections or other adverse events resulting from the transplant.

A number of countries have since introduced regulatory frameworks or guidelines for xenotransplantation. The US Food and Drugs Agency (FDA) has set out detailed and strict rules to minimize public health risks [15, 16]. These rules recognize that xenotransplantation involves standardized production of biological material that has inherent risks to the recipients, and that standards therefore need to be developed for the production and approval of such products, similar to those for pharmaceutical products. The Changsha Communiqué clearly states that government regulations “should have a legal basis with powers to ban unregulated procedures and enforce compliance with regulatory requirements.” [17] There is little disagreement about this point and general agreement about what standards should be available for the evaluation of the material or organ that is used for xenotransplantation, including in China [18]. Unlike the early 2000s, when some groups called for research moratoria, there is now less concern about adverse public health risks, provided that current guidelines for the production of xenotransplantation tissue and organs are followed.

There is therefore no lack of adequate standards, guidelines, or regulations for the evaluation of the public health risks of xenotransplantation. However, the crucial issue is whether existing procedures avoid the absence of conflicts of interests when these risks are evaluated. This is particularly the case if existing rules allow xenotransplantation proposals to move forward following evaluation by local experts. The main ethics worry today is to ensure that existing rules and guidelines are followed before xenotransplantation is initiated in a clinical setting, and that clinical procedures are not unduly influenced by commercial or other conflicting interests.

## 5 RISKS TO TRANSPLANT RECIPIENTS: ETHICS OF CLINICAL TRIALS

Xenotransplantation is still not an established medical practice, and therefore needs to be considered as clinical research. It is thus subject to standard clinical research ethics requirements. This means the risks to patients need to be carefully weighed against potential benefits by an independent review mechanism. Two of the risks are the possibility of immune rejection and the possibility of life‐threatening infections in the recipient. Advances in the ability to modify the genetic makeup of the animal tissue make it less likely to be rejected by the human recipient, and immune rejection is therefore less of a worry these days. Furthermore, the recent Maryland case at least demonstrated that there was no immediate, strong immune reaction to the transplanted organ. The death of the recipient may have been caused by an infection, which of course remains a general worry [19, 20]. This concern is about the viability of the transplant itself, and is separate from the worry about any public health risks as a result of any infection.

The inherent uncertainties in the risk assessments, especially given the extremely limited experience with transplants in human subjects, mean that xenotransplantation is fundamentally different from transplantation using human tissue or organs. Its status is therefore similar to Phase I drug research, and both “first in human” research and Phase I oncology trials. These trials are subject to more stringent requirements than standard Phase III trials, where there is already quite extensive evidence from previous human experiments. Notably, the US FDA only gave emergency authorization for the Maryland xenotransplantation experiment. It did not conclude that there was enough evidence for a more general authorization. The special nature of clinical xenotransplantation research means that ethical review needs to consider several particular issues.

### 5.1 The choice of patients

It is generally accepted that the inclusion of potential subjects in xenotransplantation clinical trials should be based on scientific criteria (such as medical standards, adequate function of other organs, negative screening for infectious diseases, and surgical standards). These criteria are similar to the clinical criteria for allogeneic transplantation. However, there are important differences between the ethical standards in clinical trials of xenotransplantation and other transplantation. Similar to oncology Phase I research, only patients who have exhausted other therapeutic options and are likely to die because there are no other established effective treatments should be included in early xenotransplantation clinical trials. These patients cannot obtain allogeneic human organs or mechanical devices for some reason [12, 21].

There are also affordability criteria. If the healthcare system dictates that patients must pay for medical care for allogeneic transplants, but care is free in trials of xenotransplantation, then individual patients may be recruited to xenotransplantation trials because they cannot afford an allogeneic transplant. This could mean that patients are recruited to xenotransplantation trials simply for reasons of economic deprivation, and not because they satisfy the independently required scientific and ethical criteria.

There may also be problems with studies among children. Early clinical trials of xenotransplantation will be a therapeutic trial and a study. Therapeutic research must focus on the prospect of benefiting patients. Clinical trials of xenotransplantation involve significantly more uncertainty than conventional treatments. It is therefore difficult to justify the inclusion of children if the same results can be achieved in trials among adults [21].

Similarly, there are issues around recruitment of people who are incapacitated or have restricted capacity. If a therapeutic trial is the most beneficial option for incapacitated adults, it might be reasonable to allow them to participate. However, we lack sufficient evidence of benefit in early xenotransplantation clinical trials, and adults lacking full capacity should therefore not be included. Similarly, there should be strict conditions for recruiting people with limited capacity. Individuals with limited capacity should be recruited to early clinical trials only when there is evidence to support the safety of progressing from animal studies to human trials and that participation in the trial will benefit the individuals concerned.

### 5.2 The issue of individual autonomy

To respect the ethical requirements of patient autonomy, it is necessary to consider whether participating in xenotransplantation trials is in line with their own medical goals and values, and in their best interest. Both international and domestic guidelines require that patients' informed consent is a necessary condition for inclusion in xenotransplantation clinical trials. However, informed consent only meets the “necessary condition” requirement, and is not a “sufficient condition.” Consideration of whether there is an acceptable risk–benefit ratio in the proposed trial is the primary responsibility of the researcher.

International and domestic ethics standards stipulate that research participants must be able to withdraw from the research at any time without any reason, and withdrawing from the research will not affect the quality of the medical services that they receive [22, 23]. To protect the public from xenotransplantation‐induced viral infection or other possible unknown risks, strict tracking and restrictions on participation are required by many of the international guidelines [12, 13, 17, 18]. Therefore, at a minimum, before the start of the clinical trial, the participants must be informed about this and their informed consent must be obtained in advance, rather than after the fact. However, it is questionable whether it is possible to give meaningful consent to life‐long monitoring because international research ethics guidelines require participants to have an independent right to withdraw for any reason at any time. This is an unresolved issue in the research ethics of xenotransplantation, and agreement must be reached about how to deal with this challenge.

The Nuffield council report in the UK states: “It would hardly be acceptable to isolate xenograft recipients suffering from an infectious disease, or to ask them to refrain from sexual intercourse or, in the case of a virus transmitted from parent to offspring, from having children” [12]. The report made clear that such restrictions would be ineffective and ethically questionable. However, it also states: “Since the possible consequences of developing xenotransplantation are potentially very serious, the principle of precaution should apply. This requires that action is taken to avoid risks in advance of certainty about their nature” [12]. The argument seems to be that xenotransplantation cannot be performed without certainty about the safety of the public [1]. If it is determined to be safe for the public, then no tracking restrictions are required. This is the heart of the dilemma.

Should clinical trials be conducted only after safety issues have been completely resolved? However, the safety of the public can only be established through careful clinical trials. If clinical trials are conducted, should there be long‐term follow‐up of patients undergoing xenotransplantation trials and restrictions put in place on them and on practice to protect the safety of the public? If measures should be taken, how long should the restrictions last? Are recipients allowed to donate blood or give birth? There has been comparatively little discussion of these issues, either internationally or in China. More ethics research and debate are necessary.

### 5.3 Regulatory issues

Considering the risks of transplantation and the challenges of clinical trials, ethical principle of prevention is essential.

Before any clinical trials are approved or take place, a legally binding regulatory framework and regulatory mechanism should be established for the use of animal organs to control the safety and quality of animal organs (and tissues) used for xenotransplantation. It is ethically acceptable to use source animals raised under conditions where all known infectious microorganisms are monitored and controlled.

Before commencing human trials, standards and mechanisms should be developed to monitor recipients of transplant clinical trials. Plans should be drawn up to take appropriate action when the disease spreads. A xenotransplantation registry should be established to record all information about individuals receiving xenotransplants. Recipients should be regularly tested for signs of disease and associated symptoms, and xenotransplantation interventions should be suspended if there are any signs of new infectious diseases.

The ethics review committee should be composed of specialized and competent scientific, medical, ethical and related disciplinary experts. So as to be able to conduct in‐depth analysis and discussion on the ethical, social, and even philosophical issues arising from xenotransplantation.

## 6 ISSUES RELATED TO AN EQUITABLE DISTRIBUTION OF HEALTH RESOURCES AND SOCIAL ATTITUDES

I have already mentioned that it would be unacceptable if early clinical trials only exposed relatively poor patients to the risks of experimental procedures. Given the high cost of transplantation in general, this could result in a scenario where one group of relatively poor people is exposed to the risk of development of procedures that would subsequently primarily be available only to wealthier patients. From an equity standpoint, this is clearly unacceptable. There is also the general issue that the research and development costs of xenotransplantation are relatively high, and continuous investment is required to try to solve the various problems. We should ask whether this is a justified prioritization of resources, or if scarce resources could be better used for preventive measures, or in improving current organ procurement and distribution systems.

In addition to issues of social justice, there are social attitudes to consider, relating to philosophical questions about human identity and integrity. In the process of development and application of advanced life technology, there is a growing tendency to objectify people. Xenotransplantation has a potential impact on the identity and integrity of the individual, and also challenges the fundamental integrity and intrinsic value of people in general. It can be seen as a further “dehumanization” or “artificialization” of people and their bodies.

Xenotransplantation does not mean it is a human–pig chimera. It still means that there are boundaries between species. Even if these technologies may be acceptable and we encourage the development of this technology because of the potential for human benefits, we still need to take a cautious stance. A rapid adoption process that does not take into account existing social and cultural worries may risk a backlash, especially if adverse events occur. There are numerous examples in the history of medicine of brilliant individuals who have faced this type of backlash, from historical giants such as Robert Koch to more contemporary examples, such as the surgeon Paolo Macchiarini, once a celebrated transplant surgeon at the Karolinska Institute in Sweden, who was recently convicted of causing bodily harm to a patient [24]. The scandal led to the resignation of two members of the Nobel Prize Assembly in Sweden because they had not intervened earlier. It is therefore also in the self‐interest of scientists to proceed carefully when adopting new technologies.

## 7 CONCLUSIONS

Moving forward with xenotransplantation is a significant scientific exploration. However, there is still a long way to go to achieve the leap from animal experiments to clinical applications, and then to safely realizing the complex functions of human organs such as the heart. The FDA review process is very strict: David Bennett's xenotransplantation trial was conducted under the “compassionate use” clause of the US FDA, and xenotransplantation has never been officially approved in the United States. The reasons for caution include the serious scientific, ethical, and social challenges, as well as hidden dangers that may have catastrophic consequences for public health. These remind us to keep an open mind.

It is also impossible for xenotransplantation to completely solve the demand for allogeneic human organs in the short term. Even with the continuous accumulation of scientific understanding and technological progress, it will only be one option among many for a considerable period of time in the future. It will therefore remain very important to demonstrate to potential and actual human organ donors that their donation is an important part of viable treatments and that its value is not diminished in any way. For the foreseeable future, society will continue to need donated human organs. The promotion of human organ donation (such as organ donation after the death of citizens) cannot be relaxed. A realistic and pragmatic approach to addressing transplantation services should strive to increase the number of allogeneic human organs available for transplantation. As at least one commentator has said, if there are organs of the same species available for transplantation, most of us would prefer to use those instead of animal organs.

Xenotransplant researchers, the media, and all those who influence public opinion have a responsibility to ensure that reporting on the progress of xenotransplantation is as accurate and objective as possible. The driving force behind the development of xenotransplantation should focus on addressing unmet health needs, rather than pursuing scientific innovativeness for its own sake [1]. It is necessary to keep a cool head about xenotransplantation, and not to rush into clinical trials when the conditions are not ripe. The Campaign for Responsible Transplantation (CRT), a civil organization composed of experts, scholars, and members of the public in the United States, mainly discusses the ethical, legal, and social problems in organ transplantation. It has raised many questions about the xenotransplantation research being conducted by many biotech companies, criticizing the idea that advances in xenotransplantation can only be achieved by clinical trials. It also pointed out that research by biotech companies is primarily driven by the enormous economic benefits of selling expensive immunosuppressants, breeding genetically modified pigs, and reaping billions of dollars, rather than providing patients hope to relieve pain [13].

We should be alert to the coercion of capital, adhere to the ethical requirements of promoting innovation and preventing risks, and ensure that science and technology is used for social good.

## AUTHOR CONTRIBUTIONS

**Xiaomei Zhai**: conceptualization (equal); formal analysis (equal); writing – original draft (equal); writing – review & editing (equal).

## CONFLICT OF INTEREST

The author declares no conflict of interest.

## ETHICS STATEMENT

None.

## INFORMED CONSENT

None.

## Data Availability

Data sharing not applicable to this article as no datasets were generated or analyzed during the current study.
